# ED Assembly: Introducing a Simple Method of Bringing Emergency Department Staff Together to Facilitate Improvement; A Report of a Real Experience

**DOI:** 10.22114/ajem.v0i0.212

**Published:** 2019-07-28

**Authors:** Asim Nayeem, Mark Hinchcliffe, Katherine Gumbs

**Affiliations:** Ashford & St Peter’s Hospitals NHS Foundation Trust, Chertsey, Surrey, UK.

**Keywords:** Assembly, Emergency Department, Overcrowding, Quality Improvement, Team Work

## Abstract

**Introduction:**

The emergency department (ED) at Ashford and St Peter’s Hospitals NHS Foundation Trust (ASPH) is a medium size department which sees around 260–300 patients per day. As a result of sustained demand, we continue to struggle to meet the four hour waiting target and face similar challenges of those of ED’s nationally. Working in a busy ED is challenging and demanding. specific challenges around communication and risks arise directly from the unique contextual demands of the ED environment.

**Objective:**

Aim being to improve the productivity of the ED team and find a mechanism to create a more supportive and enjoyable working environment within the department.

**Method:**

Our clinical leadership started looking for answer to improve communication among team members and to create a platform where there was no hierarchy and all team members could be directly involved in problem solving. With the support of the quality improvement (QI) team, ED assembly was born.

The assembly is a simple method of regularly bringing together staff to facilitate improvement and better team working. It is a platform for effective communication and innovation, in which there is no hierarchy and everyone is encouraged to contribute.

**Results:**

The assembly runs to a routine; every other Wednesday at 11^am^, the team come together for just half an hour. The agenda is set by the team in advance and everyone is encouraged to contribute their ideas and items they wish to contribute to others. Here are some examples of the quality improvement initiatives that have been born out of ED assembly: ED board rounds, coding information, overdose proforma, timely completion of standard investigations, access to fracture clinic appointments, nil-by-mouth communication, safety huddles, patient safety and sepsis, inclusive improvement, adoption of the ED assembly model by other teams and etc.

**Conclusion:**

ED assembly has supported many small but effective QI initiatives and regular communications support timely feedback on progress and update on plan-do-study-act (PDSA) cycles, resulting in changes in the everyday practice and improved pathways of patient care.

## Introduction

Emergency department (ED) serve as the front line of care for the injured and severely ill. The pressure on the UK’s EDs has been described by the Royal College of Emergency Medicine as the “biggest operational challenge facing the national health system (NHS)” as more and more people are attending, and it is stretching the ability of departments to cope (1).

Working in a busy department is both challenging and demanding; communication can be particularly difficult, and risks arise directly from the unique contextual demands of the ED environment. Shortage of staff at every level across the NHS is hitting every hospital and the effects are being felt more severe in EDs where recruitment and retention can be difficult, due to the demanding nature of the role. A steady increase in attendances, a falling number of doctors and nurses, waiting time target pressure and poor work/life balance is resulting in bottlenecks throughout the hospital system that back up into the ED; “With bed occupancy high, staff under strain and resources tight, there is no doubt that the system is near breaking point” (2).

The ED at St Peter’s Hospital is a medium size department, seeing around 260–300 patients per day. As a result of sustained demand, meeting the 4-hour waiting target continues to be a struggle so is there anything we can do to try to alleviate this problem and improve staff morale in ED?

At ASPH, there is excellent setup with a dedicated quality improvement (QI) team working under the umbrella of ‘Be the Change’; the trust-wide program and mechanism for identifying, supporting and affecting staff-led improvement projects. The QI team supports individuals and teams to be creative and innovative, providing help to continue to find ways, in which services and care can be improved, their work is supported by the executive team and hierarchy.

One of the biggest challenges in any ED is communication, hence we set out to find a way to provide a platform for the team that would allow them to directly input into decisions that influence the department and given them the freedom to raise ideas for improvement, regardless of their position within the team hierarchy.

We searched the options available; concept of daily clinical huddles, daily meetings as seen in motor industry, possibility of monthly debrief sessions outside work environment, frequent quality and safety day type events, offer platform for anonymous feedback, but it was felt that none of the above fit the purpose in a busy ED.

In September 2015, some members of the QI team and Dr Nayeem visited Sheffield Teaching Hospitals NHS Foundation Trust where there is a Microsystems Coaching Academy program, which supports staff to carry out QI work, through training and team coaching. As part of the visit, the team attended a ward improvement meeting and witnessed an example of structured, well-led, non-hierarchical meeting. We decided to use the model seen in Sheffield with changes needed to implement the model in a busy ED. Aim being to improve the productivity of the ED team and find a mechanism to create a more supportive and enjoyable working environment within the department.

## Methods

With the support of the ASPH QI team, ED took the core concept of what they saw at Sheffield and created an effective meeting with the aim to provide staff with a platform of no hierarchy, an opportunity to lead improvement projects, to support and provide tools to lead changes with the aim to have a huge amount of work completed within a short (30 minutes) time via an effective ED meeting called ‘ED assembly’.

ED assembly is a simple method of bringing staff together to facilitate improvement and better team working. It is a platform for effective communication, in which there is no hierarchy and everyone is encouraged to contribute. The whole ED team is invited; in fact, anyone from across the Trust is invited. The assembly creates time and space for the whole team (doctors, nurses, porters, housekeepers, administrative staff and managers) to come together. It runs to a routine, every other Wednesday at 11^am^ for half an hour.

The agenda is set by the team in advance and everyone is encouraged to contribute their ideas and any items they wish to communicate to others. There is a timekeeper and note taker allocated for each meeting and short term actions are agreed. It is important that progress is made from one meeting to another in order to generate the rapid improvement cycles that create the energy to fuel the continued desire for staff to improve and find better ways of working. Since the inception of ED assembly, there has been multiple improvement projects completed resulting in improved quality of care and safety for patients attending the ASPH ED.

## Results

Here are some examples of the quality improvement initiatives that have been born out of ED Assembly

### ED board rounds

ED board rounds were sporadic and at times, poorly attended. As a result of discussion at ED assembly and actions taken via this meeting, the consistency of and attendance at ED board rounds have been much improved; they are in place four times a day and are attended by the whole team

### Coding information

The capturing and recording of vital coding information in patient notes are important to ensure correct recording of patients’ presenting complaints, discharge information and for accurate funding for the hospital. The ED team are often the only contact for a patient if they are not admitted hence precise coding is very important. ED assembly was used to highlight the importance of this by having coding as a standing agenda item for a number of weeks and improvement has been seen as a result. This demonstrates that the assembly is a useful vehicle for communication of important messages to the whole team

### Overdose proforma

A proforma to provide essential guidance on the management of patients attending ED following an overdose, was devised in conjunction with specialist nurses and successfully introduced following a discussion at ED assembly. This has improved the screening information that is captured for these patients and helped to give staff confidence when asking some difficult questions

### Timely completion of standard investigations

Nursing staff raised concerns at an ED Assembly meeting regarding the timely completion of initial patient investigations (electrocardiograms, Venus blood gas analyses). Doctors and health care assistants responded to these concerns and ensure that these basic tests are completed on arrival and are checked and signed by a doctor immediately post triage.

### Access to fracture clinic appointments

Availability and access to fracture clinic appointments is a particular problem in the winter months. Joint working with the fracture clinic has resulted in increased capacity for ED patients to refer straight to the fracture clinic by 25% in winter months.

### Nil-by-mouth communication

As ED is a busy environment with a high turnover of patients, it is very important that any specific needs of individual patients are communicated clearly to the whole ED team. Improving the communication of ‘nil-by-mouth’ was raised as a topic in ED assembly by catering staff following an incident when they fed a patient awaiting surgery by mistake. As a result of the ED assembly discussion, identification cards were introduced for patients who were nil-by-mouth and a team was created to ensure that the cards are being used. This has resulted in reduced chances of errors, as previously staff had to communicate verbally regarding nil-by-mouth orders.

### Safety Huddles

ED is a busy and complex environment and it carries unique risks. This topic was discussed at ED assembly following an unfortunate incident when due to poor communication between staff members; the deterioration of an elderly patient admitted to our clinical decision unit (CDU) was not conveyed to senior clinical staff. A team was selected to look for solutions to improve patient safety and communication in CDU and introduced ‘safety huddles’ within CDU and the urgent care centre (UCC). Safety huddles take place at a regular time twice daily and they aim to provide a no-fear no-blame, safe environment in the daily work of staff. The team members use the huddles to discuss any issues of the day that may affect patient safety as well as provide an opportunity to update and act on ‘live’ safety concerns, enabling mitigating actions to be put in place very quickly.

### Patient safety and sepsis

Sepsis is recognised as a significant cause of mortality and morbidity in the NHS with around 32,000 deaths in England attributed to Sepsis annually. Of these, some estimates suggest 11,000 could have been prevented (3). Through ED assembly, the sepsis working group works with the ED team to achieve improve screening and treatment of patients for sepsis attending the ED; a key quality objective of the organisation. ED and the QI team designed a sepsis proforma that is being used in the ED. Through ED assembly, the team worked to embed the use of the proforma, respond to feedback on the proforma and discuss further ideas. Over a year, the team in ED improved the screening of patients for Sepsis to over 95% and increased the percentage of patients receiving antibiotics within one hour of arrival to ED to 90%.

### Inclusive improvement

ED assembly provides a unique opportunity for clinical and non-clinical team members to come together and work to improve their daily working practices. One example is how our porters and nursing teams were able to discuss the frequent problems they faced with communication and availability at busy times, a small improvement team was formed to identify ways to improve communication between clinical staff and porters. The team created a porters’ station in the main department and introduced an electronic system to request jobs. This has improved communication and team working. This demonstrates how ED assembly empowers team members from a diversity of roles to work together to deliver inclusive improvement.

### Adoption of the ED assembly model by other teams

We encourage attendance at the assembly meetings from all Trust staff. As word has spread about its success, other teams have wanted to try it out for themselves and have gone ahead with our support and started their own assemblies. The QI team provide support to other departments in setting up meetings within their areas; consequently, we now have a Neonatal Assembly, Paediatric ED Assembly, and an Acute Medical Unit Assembly held every fortnight.

## Discussion

At ASPH, we aim to empower all staff to identify quality improvement opportunities in their own areas and support them to make these improvements themselves. The ED assembly has not only enabled all ED staff to come forward with ideas, but has actively encouraged the team to work together to make improvements and to develop new skills in doing so. As well as making improvements to individual processes and pathways, ED assembly aims to improve the environment for staff and patients alike. There is a well-established relationship between staff experience and patient experience (4). ED assembly is one way that the staff are improving the teamwork and culture in the department. Facilitating teamwork, improvement and respect means staff are more likely to raise concerns or ask, which helps to improve safety.

Our chief executive at ASPH, said that “we are embracing QI, not only because it is the right thing to do, but because it will help us achieve the culture of curiosity and creativity where we all feel empowered and confident in looking for improvements for the benefits of our patients.” ED assembly is testament to this developing culture of curiosity and creativity and demonstrates what can be achieved by well supported and well led staff in a short space of time.

## Conclusions

ED assembly has supported many small but effective QI initiatives and regular communications support timely feedback on progress and update on plan-do-study-act (PDSA) cycles, resulting in changes in the everyday practice and improved pathways of patient care.
